# Bacteriophage and Fusidic Acid Have Synergistic Effect Against Meticillin‐Resistant *Staphylococcus pseudintermedius* in Ex Vivo Canine Dermis Model

**DOI:** 10.1111/vde.70030

**Published:** 2025-09-18

**Authors:** Sarah Ehling, Elisa Peh, Hilke Oltmanns, Jessica Meissner, Sophie Kittler, Christina Puff, Jutta Verspohl, Mathias Müsken, Madeleine Plötz, Andrea Vanessa Volk

**Affiliations:** ^1^ Department of Dermatology, Small Animal Hospital University of Veterinary Medicine Hannover Foundation Hannover Germany; ^2^ Institute for Food Quality and Food Safety University of Veterinary Medicine Hannover Foundation Hannover Germany; ^3^ Department of Pharmacology, Toxicology Und Pharmacy University of Veterinary Medicine Hannover Foundation Hannover Germany; ^4^ Department of Pathology University of Veterinary Medicine Hannover Foundation Hannover Germany; ^5^ Institute for Microbiology University of Veterinary Medicine Hannover Foundation Hannover Germany; ^6^ Central Facility for Microscopy Helmholtz Centre for Infection Research – HZI Braunschweig Germany

**Keywords:** bacteriophage (phage), canine ex vivo dermis model, Franz diffusion cells, meticillin‐resistant *Staphylococcus pseudintermedius*

## Abstract

**Background:**

Antimicrobial stewardship has become vital given the progressive emergence of multidrug‐resistant bacteria, and novel approaches to the treatment of bacterial infections are needed. Recently, reported synergistic effects of antibacterial drugs and bacteriophage therapy have revealed promising applications for the management of meticillin‐resistant staphylococcal infections.

**Objectives:**

The objective of this study was to investigate the response of meticillin‐resistant *Staphylococcus pseudintermedius* (MRSP) to treatment with a newly isolated, lytic MRSP‐specific bacteriophage. Furthermore, a postulated synergism between phage and fusidic acid was examined in a canine ex vivo dermis model.

**Material and Methods:**

Skin was harvested from the lateral thorax of a euthanised dog, clipped, the subcutis removed, and epidermis cleaved via a modified salt‐split technique. The ex vivo dermis model established in Franz diffusion cells was inoculated with 1 × 10^7^ colony‐forming units (cfu) of a clinical MRSP strain for 16 h. Then, experimental groups were treated with phage vB_SpsS_LmqsKl44‐4 at a concentration of 2 × 10^6^ plaque‐forming units and fusidic acid 0.4 mg alone or in combination for an additional 8 h.

**Results:**

Histopathological results showed that colonies of MRSP reached the superficial dermis and entered hair follicles. Co‐treatment with fusidic acid and phage significantly reduced the amount of MRSP after 8 h.

**Conclusions and Clinical Relevance:**

In conclusion, topical co‐treatment with fusidic acid and a phage could be a promising approach to the treatment of canine MRSP pyoderma.

## Graphical abstract

**Background:** Antimicrobial stewardship has become vital given the progressive emergence of multidrug‐resistant bacteria, and novel approaches to the treatment of bacterial infections are needed. Recently, reported synergistic effects of antibacterial drugs and bacteriophage therapy have revealed promising applications for the management of meticillin‐resistant staphylococcal infections. **Objectives:** The objective of this study was to investigate the response of meticillin‐resistant *Staphylococcus pseudintermedius* (MRSP) to treatment with a newly isolated, lytic MRSP‐specific bacteriophage. Furthermore, a postulated synergism between phage and fusidic acid was examined in a canine ex vivo dermis model. **Conclusions and Clinical Relevance:** In conclusion, topical co‐treatment with fusidic acid and a phage could be a promising approach to the treatment of canine MRSP pyoderma.

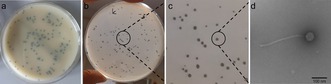

## ZUSAMMENFASSUNG

**Hintergrund:**

Die „Antimikrobielle Stewardship“ ist in Anbetracht der fortschreitenden Zunahme an Multidrug‐resistenten Bakterien absolut notwendig geworden. Es werden neue Herangehensweisen zur Behandlung von bakteriellen Infektionen benötigt. Jüngst haben synergistische Effekte von antibakteriellen Medikamenten und von Bakteriophagen Therapie vielversprechende Anwendungen zum Management Methicillin‐resistenter Staphylokokkeninfektionen gezeigt.

**Ziele:**

Das Ziel dieser Studie war es die Reaktion von Methicillin‐resistenten *Staphylococcus pseudintermedius* (MRSP) auf eine Behandlung mit neu isolierten, lytischen MRSP‐spezifischen Bakteriophagen zu untersuchen. Weiters wurde ein postulierter Synergismus zwischen Phagen und Fucidinsäure an einem caninen *ex vivo* Hautmodell untersucht.

**Materialien und Methoden:**

Es wurde Haut vom lateralen Thorax eines euthanasierten Hundes geerntet, geschoren, die Subkutis entfernt und die Epidermis mit einer modifizierten Salzspaltungsmethode gespalten. Dieses *ex vivo* Hautmodell, welches in Franz Diffusionszellen hergestellt war, wurde mit 1 x 10^7^ Kolonie‐bildenden Einheiten (cfu) eines klinischen MRSP‐Stamms für 16 h inokuliert. Danach wurden die experimentellen Gruppen mit Phage vB_SpsS_LmqsKl44‐4 bei einer Konzentration von 2 x 10^6^ Plaques‐bildenden Einheiten und Fucidinsäure 0,4 mg allein oder in Kombination für eine zusätzlich Dauer von 8 h behandelt.

**Ergebnisse:**

Die histopathologischen Ergebnisse zeigten, dass MRSP‐Kolonien die oberflächliche Dermis erreichten und in die Haarfollikel eindrangen. Eine Begleitbehandlung mit Fucidinsäure und Phagen reduzierte das Ausmaß von MRSP nach 8 h signifikant.

**Schlussfolgerungen und klinische Bedeutung:**

Zusammenfassend könnte die topische Begleitbehandlung mit Fucidinsäure und einem Phagen eine vielversprechende Herangehensweise bei der Behandlung einer caninen MRSP Pyodermie sein.

## 摘要

**背景:**

随着多重耐药细菌的不断出现，抗菌药物管理已变得至关重要，亟需新的细菌感染治疗方法。近期报道显示，抗菌药物与噬菌体疗法的协同效应在治疗耐甲氧西林葡萄球菌感染方面展现出有前景的应用。

**目的:**

本研究旨在评估一种新分离的、裂解性、针对耐甲氧西林假中间型葡萄球菌(MRSP)的特异性噬菌体的治疗效果。此外，还在犬离体真皮模型中探讨了噬菌体与夫西地酸之间可能的协同作用。

**材料与方法:**

取安乐死犬的胸侧皮肤，经剃毛、去除皮下组织，并采用改良盐分离技术剥离表皮。利用Franz扩散池建立离体真皮模型，并接种1 × 10⁷ CFU的临床MRSP菌株16小时。随后，实验组分别给予浓度为2 × 10⁶ PFU的噬菌体vB_SpsS_LmqsKl44‐4、夫西地酸0.4 mg单独或联合处理8小时。

**结果:**

组织病理学结果显示，MRSP菌落可到达浅层真皮并进入毛囊。夫西地酸与噬菌体的联合治疗在8小时后显著减少了MRSP的数量。

**结论与临床相关性:**

总之，夫西地酸与噬菌体的局部联合治疗可能成为犬MRSP脓皮症的一种有前景的治疗方法。

## Résumé

**Contexte:**

La gestion des antimicrobiens est devenue essentielle compte tenu de l'émergence progressive de bactéries multirésistantes, et de nouvelles approches thérapeutiques sont nécessaires pour traiter les infections bactériennes. Récemment, les effets synergiques rapportés des antibiotiques et de la thérapie par les bactériophages ont révélé des applications prometteuses pour la prise en charge des infections à staphylocoques résistants à la méthicilline.

**Objectifs:**

L'objectif de cette étude était d'étudier la réponse du *Staphylococcus pseudintermedius* résistant à la méthicilline (MRSP) au traitement par un bactériophage lytique spécifique du MRSP récemment isolé. En outre, une synergie supposée entre le phage et l'acide fusidique a été examinée dans un modèle de derme *ex vivo* canin.

**Matériel et méthodes:**

La peau a été prélevée sur le thorax latéral d'un chien euthanasié, tondue, le tissu sous‐cutané a été retiré et l'épiderme a été clivé à l'aide d'une technique modifiée de fractionnement au sel. Le modèle de derme *ex vivo* établi dans des cellules de diffusion de Franz a été inoculé avec 1 × 10^7^ unités formant colonie (ufc) d'une souche clinique de MRSP pendant 16 heures. Ensuite, les groupes expérimentaux ont été traités avec le phage vB_SpsS_LmqsKl44‐4 à une concentration de 2 × 10^6^ unités formant plaque et de l'acide fusidique à 0,4 mg, seuls ou en association, pendant 8 heures supplémentaires.

**Résultats:**

Les résultats histopathologiques ont montré que les colonies de MRSP ont atteint le derme superficiel et pénétré dans les follicules pileux. Le traitement combiné à l'acide fusidique et au phage a considérablement réduit la quantité de MRSP après 8 heures.

**Conclusions et pertinence clinique:**

En conclusion, le traitement topique combiné à l'acide fusidique et à un phage pourrait constituer une approche prometteuse pour le traitement de la pyodermite canine à MRSP.

## 要約

**背景:**

多剤耐性菌の斬新的な出現に伴い、抗菌薬適正使用は極めて重要となっており、細菌感染症治療に対する新たなアプローチが求められている。近年、抗菌薬とバクテリオファージ療法の相乗効果が報告され、メチシリン耐性ブドウ球菌感染症の管理に有望な応用が示されている。

**目的:**

本研究の目的は、メチシリン耐性*Staphylococcus pseudintermedius*(MRSP)に対する、新たに分離されたMRSP特異的溶菌バクテリオファージによる治療への反応を調査することであった。さらに、ファージおよびフシジン酸の相乗作用の可能性を、犬由来*ex vivo*真皮モデルを用いて検証した。

**材料および方法:**

安楽死させた犬の側胸部から皮膚を採取し、毛刈り、皮下組織を除去し、改良塩分割法を用いて表皮を分離した。フランツ拡散細胞で構築した*ex vivo*真皮モデルに、臨床MRSP株1×10^7^コロニー形成単位(cfu)を16時間接種した。その後、実験群に2×10^6^プラーク形成単位の濃度のファージvB_SpsS_LmqsKl44‐4およびフシジン酸0.4mgを単独または併用でさらに8時間処理した。

**結果:**

病理組織学的検査の結果、MRSPコロニーは真皮表層まで到達し、毛包に侵入していたことが示された。フシジン酸およびファージの併用投与により、8時間後のMRSP量は有意に減少した。

**結論と臨床的意義:**

結論として、フシジン酸およびファージの局所併用投与は、犬のMRSP膿皮症の治療に有望なアプローチとなる可能性があった。

## Resumo

**Contexto:**

O consenso de uso racional de antimicrobianos tornou‐se vital devido ao surgimento progressivo de bactérias multirresistentes, e novas abordagens para o tratamento de infecções bacterianas são necessárias. Recentemente, os efeitos sinérgicos relatados de medicamentos antibacterianos e terapia com bacteriófagos revelaram aplicações promissoras para o tratamento de infecções estafilocócicas resistentes à meticilina.

**Objetivos:**

O objetivo deste estudo foi investigar a resposta do *Staphylococcus pseudintermedius* resistente à meticilina (MRSP) ao tratamento com um bacteriófago lítico específico para MRSP recém‐isolado. Além disso, um sinergismo postulado entre o fago e o ácido fusídico foi examinado em um modelo de derme canina *ex vivo.*

**Material e Métodos:**

A pele foi coletada da região lateral do tórax de um cão eutanasiado, tosado, o tecido subcutâneo removido e a epiderme clivada por meio de uma técnica modificada de divisão de sais. O modelo de derme *ex vivo* estabelecido em células de difusão de Franz foi inoculado com 1 × 107 unidades formadoras de colônias (ufc) de uma cepa clínica de MRSP por 16 h. Em seguida, os grupos experimentais foram tratados com o fago vB_SpsS_LmqsKl44‐4 na concentração de 2 × 106 unidades formadoras de placas e ácido fusídico 0,4 mg, isoladamente ou em combinação, por mais 8 h.

**Resultados:**

Os resultados histopatológicos mostraram que colônias de MRSP atingiram a derme superficial e penetraram nos folículos pilosos. O cotratamento com ácido fusídico e fago reduziu significativamente a quantidade de MRSP após 8 h.

**Conclusões e Relevância Clínica:**

Em conclusão, o cotratamento tópico com ácido fusídico e um fago pode ser uma abordagem promissora para o tratamento da piodermite canina por MRSP.

## RESUMEN

**Introduccion:**

La optimización del uso de antimicrobianos se ha vuelto vital dada la progresiva aparición de bacterias multirresistentes, por lo que se necesitan nuevos enfoques para el tratamiento de las infecciones bacterianas. Recientemente, los efectos sinérgicos reportados entre los fármacos antibacterianos y la terapia con bacteriófagos han revelado aplicaciones prometedoras para el manejo de las infecciones por estafilococos resistentes a la meticilina.

**Objetivos:**

El objetivo de este estudio fue investigar la respuesta de *Staphylococcus pseudintermedius* resistente a la meticilina (MRSP) al tratamiento con un bacteriófago lítico específico para MRSP, recientemente aislado. Además, se examinó la posible sinergia entre el fago y el ácido fusídico en un modelo de dermis canina ex vivo.

**Material y métodos:**

Se obtuvo piel del tórax lateral de un perro eutanasiado tras rasurado, se eliminó el tejido subcutáneo y se escindió la epidermis mediante una técnica modificada de división con sal. El modelo dérmico ex vivo establecido en células de difusión de Franz se inoculó con 1 × 10⁻¹ unidades formadoras de colonias (ufc) de una cepa clínica de MRSP durante 16 h. Posteriormente, los grupos experimentales fueron tratados con el fago vB_SpsS_LmqsKl44‐4 a una concentración de 2 × 10⁻¹ unidades formadoras de placa y 0,4 mg de ácido fusídico, solo o en combinación, durante 8 h adicionales.

**Resultados:**

Los resultados histopatológicos mostraron que las colonias de MRSP alcanzaron la dermis superficial y penetraron en los folículos pilosos. El cotratamiento con ácido fusídico y fago redujo significativamente la cantidad de MRSP después de 8 h.

**Conclusiones y relevancia clínica:**

En conclusión, el cotratamiento tópico con ácido fusídico y un fago podría ser un enfoque prometedor para el tratamiento de la pioderma canina por MRSP.

## 1 Introduction

Increasing prevalence of meticillin‐resistant *Staphylococcus pseudintermedius* (MRSP) in canine patients is not only a health concern for these patients, but also a threat for human health owing to the zoonotic potential [1, 2]. The European Food Safety Authority (EFSA) identified *S. pseudintermedius* as one of the three most relevant antimicrobial‐resistant bacteria in dogs and cats, with a prevalence of about 30% [3, 4]. One of the aggravating factors of resistance is the formation of biofilms, which further reduce antimicrobial efficacy [5, 6]. Thus, in the 21st Century, antibiotic stewardship is vital, and new multimodal approaches to the treatment of bacterial infections are needed.

*Staphylococcus pseudintermedius* is the most frequent cause of canine skin infections, occurring in ≤ 92% of cases, particularly in those with superficial pyoderma [2, 7]. Deep pyoderma, while less common than superficial ones, may pose a risk for haematogenous spread and systemic bacteraemia [8]. Pyoderma is one of the main presentations leading to systemic antimicrobial use in small animal practice [9], although evidence for the efficacy of systemic antimicrobial agents is sparse [10]. Nevertheless, 96.5% of dogs diagnosed with bacterial pyoderma were prescribed systemic and/or topical antimicrobials [11]. There is good evidence that topical treatment alone can be effective in superficial pyoderma, including cases with MRSP. Previous studies looking at different topical treatments for superficial pyoderma have shown high efficacy of fusidic acid against MRSP and good penetration into the skin [12, 13]. Resistance to fusidic acid is described in staphylococci isolated from infection sites in dogs and linked to certain genetic determinants in bacteria, for example, *fusA*, *fusB*, and *fusC* mutations [14]. However, the authors of this study concluded that a correlation between increased prevalence of these genes and clinical significance remains unknown owing to very high concentrations of the topical antimicrobial on the skin [14].

Virulent or obligate lytic bacteriophages (phages) are ubiquitous viruses that infect and replicate within their specific bacterial host and result in bacterial lysis [15]. They are highly host‐specific and, unlike antibacterial drugs, do not alter the surrounding microbiome [16]. Phages can be used either for monotherapy or in combination with antimicrobials. Synergistic effects have been shown for treating infections caused by meticillin‐resistant *Staphylococcus aureus* (MRSA) [17, 18, 19]. It has been hypothesised that the development of antimicrobial resistance by bacteria is less likely when two agents with different modes of action are used in a treatment regimen, compared to single‐agent treatment [20, 21]. Therefore, it may be possible to preserve the efficacy of antimicrobial drugs for the future by using a multimodal approach [22]. In recent years, *S. pseudintermedius* ‐specific phages have been isolated and further characterised in vitro [23, 24, 25, 26, 27, 28]. Kim and Giri [24] examined two *S. pseudintermedius* phages in a biofilm assay and could show that the phages inhibited biofilm formation at low concentrations and were able to degrade mature biofilms at high concentrations. However, despite these promising results, no data have been reported on the practicality of using phages for the treatment of clinical cases.

The aim of this study was to investigate the response of MRSP to treatment with a newly isolated, lytic MRSP‐specific phage in a canine ex vivo dermis model.

## 2 Material and Methods

### 2.1 Meticillin‐Resistant *Staphylococcus pseudintermedius*

The MRSP strain 5463/1/22 originated from the wound of a dog in the authors' small animal hospital. Isolation, culture, and sensitivity testing were performed at the Institute for Microbiology at the authors' university according to Clinical and Laboratory Standards Institute (CLSI Vet01) [29]. Adjusted 0.5 McF (McFarland turbidity) accounting for 1 × 10^7^ colony‐forming units (cfu) was used in the experiments.

### 2.2 Antimicrobial Susceptibility Testing

The minimum inhibitory concentrations (MIC) for commonly tested antibiotics were determined following the standards and evaluation given in the CLSI document Vet01, 5th edn [29]. According to the results of the susceptibility tests, the isolate was oxacillin‐resistant and thus classified as MRSP (see Table S1). A PCR analysis confirmed the presence of the *mec*A gene [30]. A thermal cycling program was adapted to initial denaturation for 3 min, denaturation for 30 s, annealing for 30 s at 57°C, extension for 120 s, and final extension for 10 min. The MIC of fusidic acid was determined by preparing a stock solution of fusidic acid sodium salt (Sigma‐Aldrich Chemie GmbH). Briefly, a stock solution was prepared in distilled water, and two‐fold serial dilutions were prepared in cation‐adjusted Mueller–Hinton broth. *S. pseudintermedius* strain DSM 25714 served as a quality‐control strain. Based on these results (data not shown), 400 μg fusidic acid was chosen for treating the MRSP isolate topically in the ex vivo dermis model.

### 2.3 Phage Isolation and Plaque Assay

The phage vB_SpsS_LmqsKl44‐4 (LmqsKl44‐4) used in this study was isolated from a sink drain of the authors' small animal hospital. In brief, a swab sample was transferred to SM buffer (5.8 g NaCl, 2.0 g MgSO_4_ × 7H_2_O, 50 mL 1 m Tris, adjusted to pH 7.5, filled up with distilled water to 1000 mL) and shaken overnight at 4°C. The sample was then filtered using a 0.2 μm polyethylensulfon membrane syringe filter (Carl Roth GmbH & Co. KG) and stored at 4°C until further use. For phage isolation, the soft agar overlay technique was used as described by Steffan et al. [31] with minor modifications. The MRSP strain was streaked out on Columbia blood agar base supplemented with 5% sheep blood (Thermo Fisher Scientific Oxoid Deutschland GmbH) and incubated aerobically at 37°C overnight. Grown colonies were suspended in 10 mm MgSO_4_ and adjusted to a turbidity according to a standard of 3.0 McF. A volume of 100 μL of the bacterial suspension and 100 μL of the filtered sample were transferred to 5 mL molten 0.7% Luria–Bertani agar (Carl Roth GmbH and Co. KG), vortexed, and poured onto LB base agar with 1.5% agar. After incubation for 24 h at 37°C in an aerobic atmosphere, grown bacterial lawns were examined for plaque formation. Plaques are areas without bacterial growth that indicate phage lysis. A single plaque was chosen and purified by successively picking and plating single plaques from grown soft agar overlays. Subsequently, phage LmqsKl44‐4 was propagated using the soft agar overlay method as described above.

### 2.4 Transmission Electron Microscopy (TEM) of the Phage

A negative stain of the phage LmqsKl44‐4 was performed as described previously [32]. In brief, the phage solution was adsorbed onto a carbon film, washed twice on Tris‐EDTA buffer droplets (10 mm Tris, 1 mm EDTA, pH 6.9), and negatively stained with 2% aqueous uranyl acetate by heat‐drying on a 60 W bulb after blotting excessive liquid with a filter paper. The sample was examined by TEM using a TEM 910 microscope (Zeiss) at an acceleration voltage of 80 kV and equipped with a slow‐scan charge‐coupled device (CCD) camera. The operating software ITEM (Olympus Soft Imaging Solutions GmbH) was used to measure the head diameter and tail length. The average size was calculated from five measurements using excel (Microsoft Office).

### 2.5 Skin Specimens

**FIGURE 1:**
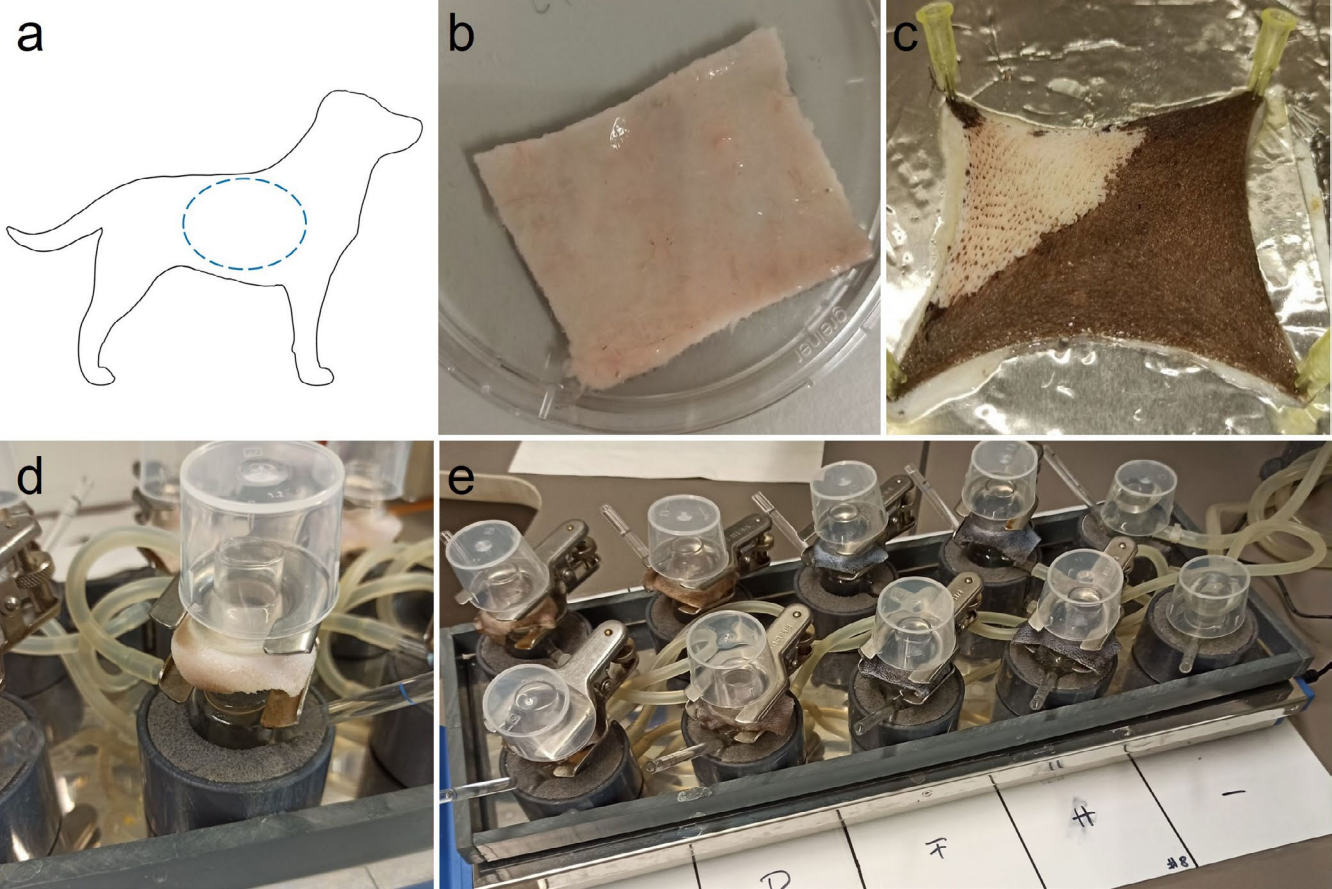
Photographs of the salt‐split technique and Franz diffusion cells experimental setup. (a) Thoracic area for sample selection. (b) Incubation in 1 m NaCl buffer subcutis facing up. (c) Pigmented skin with stratum corneum and epidermis partially removed. (d) Close‐up view of a Franz diffusion cell. (e) Parallel setup of multiple Franz diffusion cells.

Six dogs representing breeds with a predominantly telogen hair cycle, euthanised for purposes unrelated to this study (Table S2), were donated for research by the owners with informed consent. Dogs having received systemic antimicrobial therapy within the prior 72 h and/or systemic glucocorticoid therapy within the prior 4 weeks, as well as dogs with clinically altered skin, were excluded. Skin samples were harvested within 24 h of euthanasia. Hair was clipped in a 20 × 20 cm section on the lateral thorax and the skin was excised (Figure 1a). The subcutis was removed and the remaining tissue was frozen at −20°C for a maximum of 6 months.

### 2.6 Franz Diffusion Cell Ex Vivo Dermis Model

Franz diffusion cells are a well‐established model for conducting studies of skin absorption and penetration, reflected in the guidelines from the Organisation for Economic Cooperation and Development (OECD) [33]. Use of Franz cells also has been validated in previous penetration studies using split skin in one of the authors' research groups [34, 35]. Here, we expanded the use of the Franz cells to establish an ex vivo canine dermis model. The OECD guidelines also confirm the integrity of the skin for ≤ 466 days frozen or ≤ 3 days at 10°C, and also describe the use of full‐thickness skin [33].

Frozen skin samples were thawed over 4 h at room temperature. The subcutis was removed with a scalpel blade, and remaining hair was clipped. Pieces measuring 3 × 3 cm were cut, placed epidermis facing down in 1 m NaCl salt‐split solution (Figure 1b), and stored at 4°C for 44 h (adapted from De Abhishek et al. 2010 [36]). Afterwards, the detaching epidermis was carefully scraped off using a scalpel blade (Figure 1c). The specimens were incubated in phosphate‐buffered saline (PBS) for 30 min. The thickness of the specimen was determined using a calliper (Mitutoyo; data not shown). Contaminating bacteria on the skin were removed by dipping into disinfectant (Desmanol pure; Schuelke and Mayr). Cultures of pilot experiments showed that Desmanol pure was superior to ethanol 70% for removing resident skin bacteria after dipping the skin into the disinfectant five times (= 5 s) (data not shown). Re‐culture of the skin with MRSP showed confluent bacterial growth after 24 h (data not shown). After disinfection, skin samples were tapped dry and placed into the Franz cell (Figure 1d,e). The acceptor chamber was filled with sterile PBS and kept at 38°C. The skin was acclimated in the chamber for 30 min. After acclimatisation, all skin specimens in the chambers were inoculated with MRSP except for the negative controls.

### 2.7 Bacterial Inoculation and Treatment Groups

**FIGURE 2:**
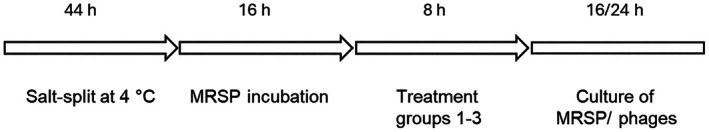
Study time‐line overview.

For each donor animal, three skin samples were prepared. All were inoculated with 1 × 10^7^ cfu of MRSP in 100 μL PBS by pipetting directly onto the skin and incubating for 16 h. Over the next 8 h, three treatment groups were established: fusidic acid 0.4 mg (Group 1) (Fucidine; Leo Pharma); phage LmqsKl44‐4 with 2 × 10^6^ plaque‐forming units (pfu) in 200 μL SM buffer (Group 2); and a combination of the two treatments (Group 3). Additionally, two negative controls were run at the same time: one with phage only, one as a complete blank (i.e., no bacteria, no phage). A timeline overview and treatment regimen are shown in Figure 2. The bacterial concentration was chosen based on previous studies [37, 38] as was the phage concentration [39, 40].

**FIGURE 3:**
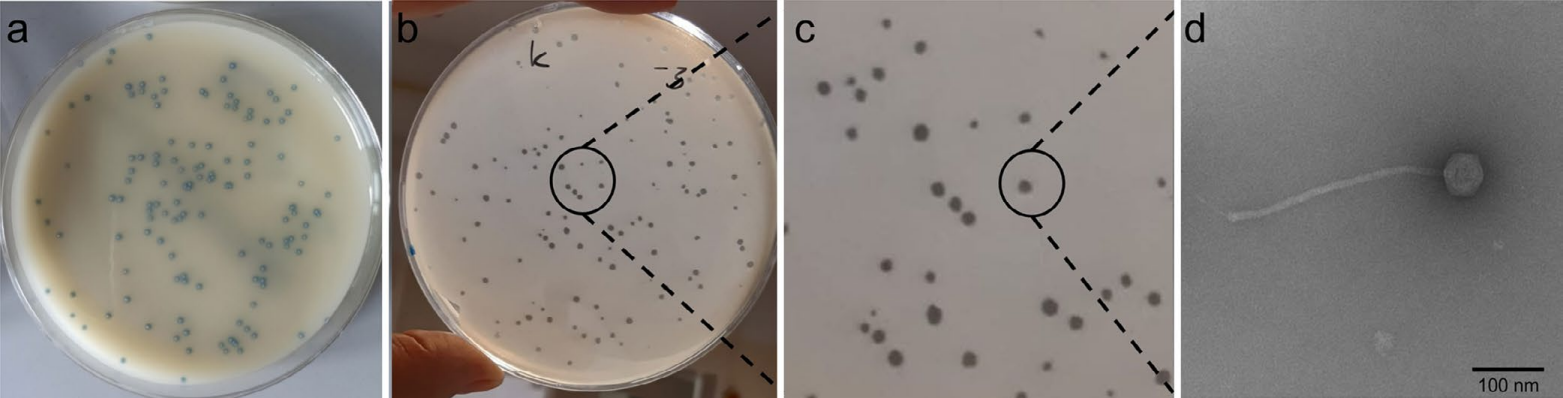
Photographs of Meticillin‐resistant *Staphylococcus pseudintermedius* (MRSP) and phage culture plates. (a) Meticillin‐resistant *S. aureus* (MRSA)‐selective agar with colonies. (b, c) Agar with lytic phage‐forming units. (d) Electron micrograph of the phage.

Treatment for groups 1, 2 and 3 was added for 8 h. The Franz cells were covered with a plastic lid to protect them against both contamination and evaporation. All steps mentioned above were performed with sterile equipment, adapted from Stahl et al. [30] After removal of the skin from the Franz cells, samples were harvested for histopathological examination (see below), extraction, and viability testing of MRSP and phage. The skin pieces were removed from the Franz cells and cut in half. One half was placed in 10% neutral buffered formalin for histopathological investigation. From the other half, an 8 mm punch biopsy was taken and placed in a 2 mL Eppendorf tube and macerated using 2 mm glass beads in 500 μL Pepton water by vortexing the tube repetitively three times for 3 s. To determine MRSP concentrations, serial dilutions up to 10^−7^ were prepared and spread out on selective agar plates in duplicates (MRSA 2 brilliance agar; Oxoid) and incubated for 16 h (Figure 3a). This agar was chosen to achieve pure cultures for MRSP [41]. The rest of the macerated tissue fluid was sterile filtered with 0.2 μm PES syringe filters for determining phage concentrations using the soft agar overlay technique as described above.

### 2.8 Histopathological Examination

In order to validate the ex vivo dermis model, the depth of penetration of the MRSP was determined in all formalin‐fixed skin samples. These were routinely processed, embedded in paraffin wax, and cut at 2–3 μm. Sections were stained with haematoxylin and eosin according to standard procedures and evaluated by a board‐certified pathologist (one of the authors) using light microscopy.

### 2.9 Statistical Methods

The Kruskal–Wallis test with Dunn's multiple comparison test was applied. Significant values are reported as: * *p* < 0.05; ** *p* < 0.01. Median and 95% confidence intervals are displayed. For the comparison of phage concentrations, for the phage‐only treatment group versus the fusidic acid plus phage group, the Wilcoxon test was applied. prism v10.4.1 software (GraphPad) was used for the analysis.

## 3 Results

### 3.1 MRSP‐Specific Lytic Phage

The isolated phage LmqsKl44‐4 formed clear plaques on the MRSP host strain (Figure 3b,c). The phage was identified as a Siphovirus. Negatively stained electron micrographs showed that the phage has an icosahedral head with a length and width of 57 nm and a tail 320 nm long (Figure 3d). In vitro synergistic interactions of fusidic acid and phage showed higher growth inhibition than single measures (see Methods S1; Figure S1; Table S3).

### 3.2 Histopathological Evaluation of MRSP Penetration in Ex Vivo Dermis Model

**FIGURE 4:**
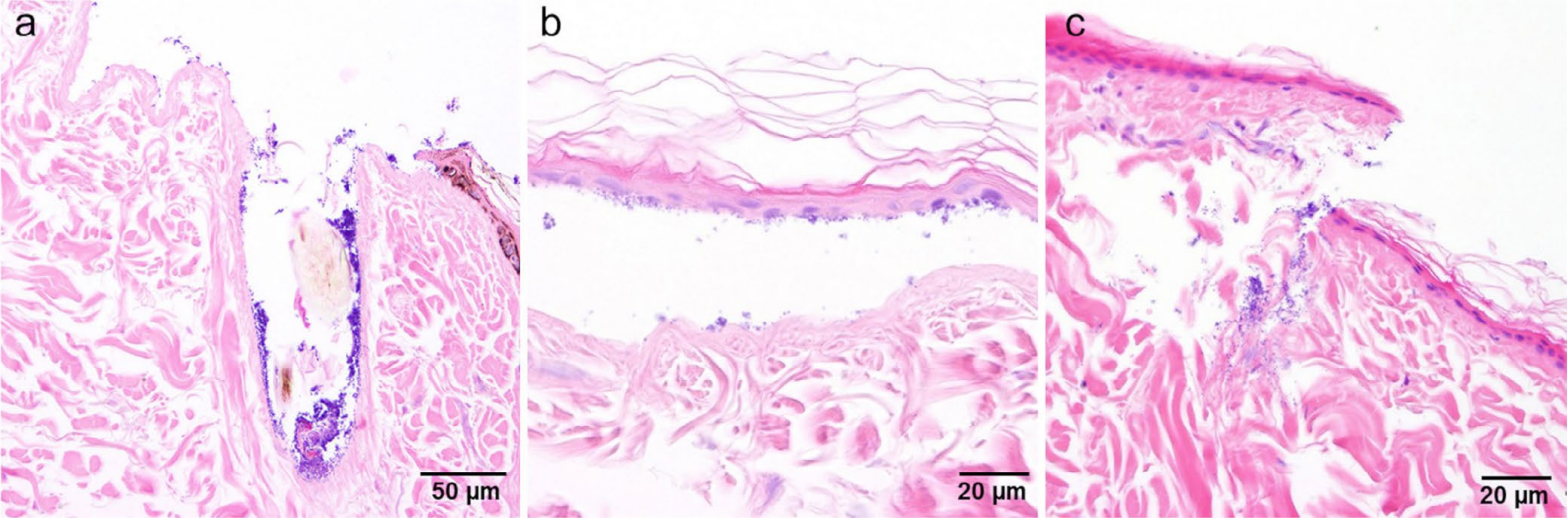
Microscopic images of stained (haematoxylin and eosin) skin sections. (a) Meticillin‐resistant *Staphylococcus pseudintermedius* (MRSP) colonisation inside the hair follicle. (b) Fissures into the dermis lead to deeper penetration of MRSP. (c) Cleft formation with MRSP attached to the bottom of the epidermis.

The canine ex vivo dermis model worked well in Franz cells with canine skin where subcutis was removed and the salt‐split technique was applied. The H&E‐stained samples showed good structural integrity of the skin after 24 h total incubation time. Histopathological evaluation showed superficial dermal colonisation of coccoid bacteria, which were also found inside the hair follicles (Figure 4a). In some samples, cleft formation resulting from the salt‐split technique revealed MRSP attaching to the bottom of the epidermis (Figure 4b). Fissures into the dermis led to even deeper penetration of MRSP (Figure 4c).

### 3.3 Treatment of MRSP With the Phage and Fusidic Acid in This Ex Vivo Dermis Model

**FIGURE 5:**
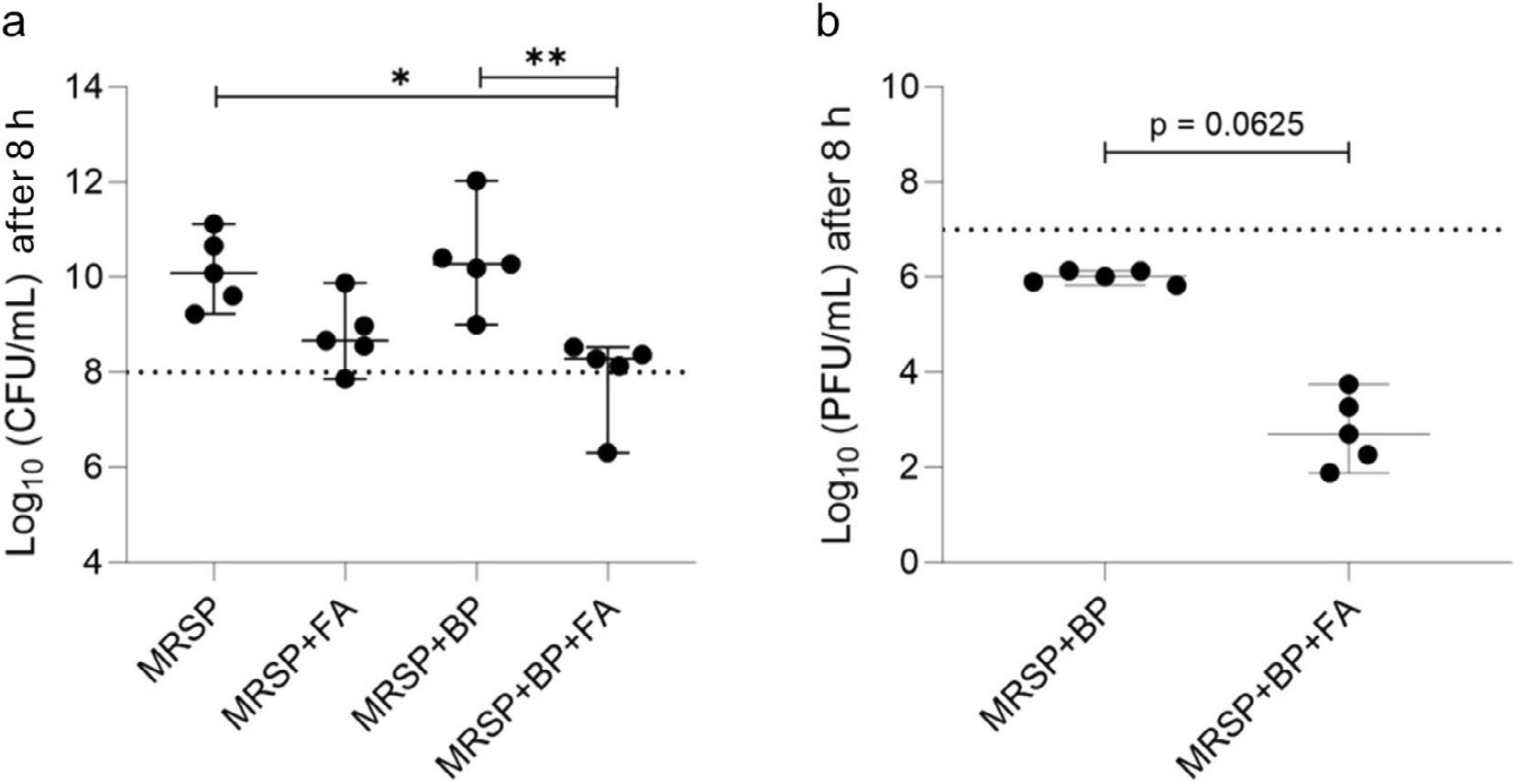
Viability testing of meticillin‐resistant *Staphylococcus pseudintermedius* (MRSP) and phage 8 h after treatment. (a) Co‐treatment with fusidic acid and phage significantly reduced the amount of MRSP after 8 treatment. (b) Phage concentrations in the group with phage and fusidic acid co‐treatment were lower than in the group with phage monotherapy. *N* = 5 dogs; median with 95% confidence interval. BP, phage; cfu, colony‐forming units; FA, fusidic acid; pfu, plaque‐forming units. Dotted line = inoculated amount of MRSP or phage at the beginning of the experiment. Significance (Kruskal–Wallis with Dunn's *post hoc* test): * *p* < 0.05; ** *p* < 0.01.

Treatment of the MRSP‐infected skin with fusidic acid for 8 h led to a reduction of MRSP (from 10.09 to 8.67 median log_10_ pfu/mL) although this was not statistically significant (*p* = 0.368). Treatment with phage alone did not change the amount of MRSP (10.28 median log_10_ pfu/mL). Nevertheless, co‐treatment with fusidic acid and phage significantly reduced the amount of MRSP after 8 h (from 10.09 to 8.29 median log_10_ pfu/mL, *p* < 0.05) compared to nontreated and bacteriophage‐treated groups (Figure 5a).

Phage concentration on the MRSP‐infected skin samples was constant after incubation for 8 (6.01 median to 7.0 log_10_ pfu/mL). When treated in combination with fusidic acid, the phage concentration showed a decrease after 8 h (from 7.0 log_10_ pfu/mL to median 2.70 log_10_ pfu/mL) (Figure 5b).

## 4 Discussion

Antimicrobial resistance in *S. pseudintermedius* presents a significant therapeutic challenge, underscoring the need for alternative or adjunctive treatment strategies. In this study, we investigated two agents with distinct mechanisms of action: fusidic acid—a bacteriostatic antibiotic that inhibits bacterial protein synthesis—and a newly isolated, lytic bacteriophage specifically targeting MRSP in an ex vivo canine dermis model.

Unexpectedly, phage monotherapy did not lead to a measurable reduction in bacterial counts. This result contrasts with both the self‐replicating nature of phages and in vitro data demonstrating their activity against MRSP. The lack of effect may be to the result of limited phage–bacteria interactions in the ex vivo tissue environment. Potential contributing factors include biofilm formation, inadequate phage diffusion, a suboptimal multiplicity of infection (MOI), and a restricted replication window. While the presence of biofilms was not confirmed, it cannot be ruled out. Phage inactivation also seemed unlikely, as viable phages were recovered postincubation. Supporting this observation, Weissfuss et al. (2023, 2025) quantified both bacteria and phages in a mouse model, using cfu and pfu to assess microbial viability [42, 43]. Phage activity is influenced by MOI; we used a relatively low MOI of 0.2 in this study to assess potential synergy with fusidic acid, rather than to maximise the efficacy of phage monotherapy. Previous studies suggest that MOIs ≥ 1—equal phage and bacteria concentrations—with phage titres of ≥ 10^8^ pfu/mL, are required for optimal bacterial adsorption and killing [39, 40]. Despite the subtherapeutic MOI, combination therapy with fusidic acid and phages significantly reduced MRSP levels, indicating a synergistic interaction.

Fusidic acid alone also reduced MRSP counts, consistent with previous reports of its bacteriostatic activity via inhibition of protein synthesis [44]. However, the effect did not reach statistical significance, possibly as a consequence of the short incubation period of 8 h. In clinical practice, topical fusidic acid is typically applied every 12 h over a course of ≥ 7 days, which may yield more pronounced effects [45].

The combination treatment of phage and fusidic acid led to a significant reduction of MRSP on the skin, consistent with the concept of phage–antibiotic synergy (PAS). PAS occurs when concurrent administration of phages and antibiotics enhances bacterial killing beyond what either agent achieves alone [20, 21, 42, 43]. Although fusidic acid is known to block protein synthesis, it remains unclear whether it increases MRSP susceptibility to phage lysis—potentially by modifying bacterial surface receptors or disrupting stress‐response pathways [44]. Interestingly, the phage concentration declined over the 8 h incubation period, despite the expectation that phages replicate within bacterial hosts before inducing lysis [46]. This could be explained by several mechanisms. One possibility is “auto‐dosing,” where a small number of phages replicate across multiple bacterial generations to achieve bacterial clearance [47], wherein phage concentrations fluctuate in relation to bacterial host density [7, 21]. Alternatively, passive lysis—killing without phage replication—may have occurred as described previously [48]. Early bacterial killing through synergistic action, phage inactivation or nonspecific binding to damaged or dying bacterial cells could have limited the number of replication cycles, thereby reducing overall phage titres. Sublethal bacterial stress or altered metabolism might have enhanced phage attachment or accelerated lysis, even without productive replication. While the short replication time of 8 h has been discussed previously, it is noteworthy that a recent study [42] demonstrated early in vivo effects of phages—both alone and in combination with meropenem—at 4 and 8 h postinfection with *Pseudomonas aeruginosa* in a mouse model of ventilator‐associated pneumonia.

In addition to interactions with antibiotics, the physical properties of phages—including capsid size and morphology—are likely to influence their ability to penetrate tissue [49]. The relatively large phage size used in this study may have resulted in limited dermal diffusion, although surface‐associated bacteria may still have been exposed. To deliver a sufficiently high phage concentration at the site of infection, a specific galenic formulation of topical phage application will play an important role in treating pyoderma in the future [40, 50]. Furthermore, phage cocktails have demonstrated superior efficacy in treating bacterial infections and reducing the likelihood of resistance developing [15, 51, 52], although the underlying mechanisms are not yet fully understood [53]. Future experiments should incorporate phage cocktails and compare their efficacy to phage monotherapy. For our proof‐of‐concept study, as described previously [52], a single phage was selected in this setting.

In order to facilitate dermal colonisation by MRSP, we modified canine skin samples using an adapted Franz cell setup. These cells are traditionally used with intact split‐thickness skin to assess transdermal drug penetration, with the stratum corneum (SC) and epidermis acting as essential barriers [33]. Therefore, modifying the established Franz cell model by removing the SC or epidermis was necessary to simulate dermal infection. Tape‐stripping [54, 55] and cyanoacrylate methods [56] were rejected owing to inconsistent removal of the SC (data not shown). Instead, we adapted the salt‐split technique, commonly used to create dermo‐epidermal clefts for immunohistochemical analysis [36]. Following cleft formation, we removed the epidermis mechanically. Histological analysis revealed that MRSP penetrated through the superficial dermis and hair follicles, yet did not reach deeper dermal layers. Extended incubation beyond 16 h may improve bacterial penetration, yet tissue degradation observed after prolonged salt‐split treatment limited this approach in our current setup (data not shown).

Interestingly, MRSP frequently adhered to the basal surface of the epidermis when splits were present. This behaviour may be mediated by adhesion molecules on deep keratinocytes or corneocytes [57, 58], which are absent in the dermis, and warrants further investigation, particularly in clinical biopsy specimens. To minimise variation, we included only short‐coated dog breeds, which typically exhibit telogen‐phase hair cycles. This contrasts with breeds such as poodles, where ≤ 98% of follicles are in the anagen phase. Hair cycle phase influences skin morphology and drug penetration; thus, breed selection helped standardise experimental conditions [34, 59].

One limitation of the study was the small sample size. One dog was excluded owing to unusually low MRSP levels in the control group, potentially resulting from undocumented prior antimicrobial exposure. Deeper bacterial inoculation and extended treatment durations may be improved if the salt‐split technique could be replaced. Additionally, phages were not visualised in the tissue sections. The size of the applied phage may have limited dermal penetration, although it may have reached bacteria on the exposed epidermal surface. Advanced imaging techniques, such as gold‐tagged electron microscopy, could help verify phage location in future studies.

## 5 Conclusions

In the context of rising antimicrobial resistance, innovative strategies are urgently needed to combat bacterial skin infections. Our study demonstrates that topical phage–antibiotic combinations may offer a promising approach to reduce reliance on systemic antibiotics, thereby potentially minimising environmental contamination and slowing the emergence of antibiotic resistance [8].

## Author Contributions

**Sarah Ehling:** conceptualisation; data curation; formal analysis; writing – draft and review. **Elisa Peh:** conceptualisation; data curation; formal analysis; writing – review and editing. **Hilke Oltmanns:** conceptualisation; writing – review and editing. **Jessica Meissner:** conceptualisation; data curation; formal analysis; writing – review and editing. **Sophie Kittler:** conceptualisation; data curation; formal analysis; writing – review and editing. **Christina Puff:** conceptualisation; data curation; formal analysis; writing – review and editing. **Jutta Verspohl:** formal analysis; writing – review and editing. **Mathias Müsken:** formal analysis; writing – review and editing. **Madeleine Plötz:** writing – review and editing. **Andrea Vanessa Volk:** conceptualisation; data curation; formal analysis; writing – review and editing.

## Conflicts of Interest

The authors declare no conflicts of interest.

## Supporting information


**Figure S1:** Growth curves of meticillin‐resistant *Staphylococcus pseudintermedius* (MRSP) with phage monotherapy or in combination with fusidic acid. Positive control: pure medium; fusidic acid at two different concentrations (miniumum inhibitory concentration [MIC] 0.5 and 0.25); phage LmqsKl44‐4 at two different concentrations (multiplicity of infection [MOI] 1 and 0.1). *N* = 1; OD, optical density.

**Methods S1:** In vitro synergy testing of fusidic acid and phage on meticillin‐resistant *Staphylococcus pseudintermedius* growth inhibition.

**Table S1:** Characteristics of the study animals.

**Table S2:** Antimicrobial sensitivity test results of the clinical meticillin‐resistant *Staphylococcus pseudintermedius* . I, intermediate; R, resistant; MIC, minimum inhibitory concentration; S, sensitive.

**Table S3:** Growth reduction (%) of meticillin‐resistant *Staphylococcus pseudintermedius* by phage monotherapy or in combination with fusidic acid. MIC, minimum inhibitory concentration; MOI, multiplicity of infection; OD, optical density.

## Data Availability

The data that support the findings of this study are available on request from the corresponding author. The data are not publicly available due to privacy or ethical restrictions.
