# Supplementary figures and images for: Bacteriophage and Fusidic Acid Have Synergistic Effect Against Meticillin‐Resistant Staphylococcus pseudintermedius in Ex Vivo Canine Dermis Model
Source: Vet Dermatol. 2025 Sep 18;37(2):200–10. doi: 10.1111/vde.70030 (PMC12967880; doi:10.1111/vde.70030)

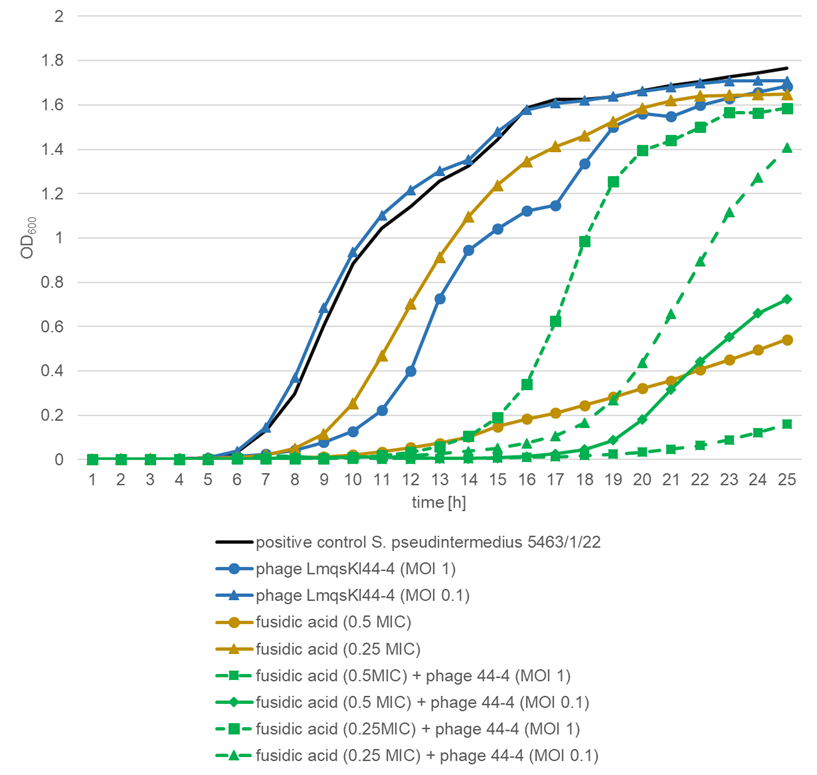

Supplement: Supplementary file 1 — Figure S1: Growth curves of meticillin‐resistant Staphylococcus pseudintermedius (MRSP) with phage monotherapy or in combination with fusidic acid. Positive control: pure medium; fusidic acid at two different concentrations (miniumum inhibitory concentration [MIC] 0.5 and 0.25); phage LmqsKl44‐4 at two different concentrations (multiplicity of infection [MOI] 1 and 0.1). N = 1; OD, optical density. [file VDE-37-200-s002.tif]
