# Supplementary material for: Bacteriophage and Fusidic Acid Have Synergistic Effect Against Meticillin‐Resistant Staphylococcus pseudintermedius in Ex Vivo Canine Dermis Model
Source: Vet Dermatol. 2025 Sep 18;37(2):200–10. doi: 10.1111/vde.70030 (PMC12967880; doi:10.1111/vde.70030)
Supplement: Supplementary file 2 — Methods S1: In vitro synergy testing of fusidic acid and phage on meticillin‐resistant Staphylococcus pseudintermedius growth inhibition. [file VDE-37-200-s004.docx]

Suppl. Methods 1:

***In-vitro* synergy testing**

For testing possible synergistic interactions of fusidic acid and phage LmqsKl44-4, growth inhibition of the MRSP was investigated via optical density (OD) absorbance measurements at 600 nm (OD_600_) using a Tecan Spark automatic microplate reader (Tecan Austria GmbH, Grödig, Austria). A 96 well microtiter plate with flat bottom was used. All experiments were conducted once. Phage suspensions were diluted to concentrations that yield multiplicity of infections (MOI) of 1 and 0.1 compared to the bacterial counts. Stock solutions of fusidic acid were prepared to obtain subinhibitory concentrations of 0.5 and 0.25 MIC in the wells of the microtiter plate. Phage and fusidic acid were tested both alone and in different combinations (Suppl.Fig.1).

To improve the comparability of the results, the area under the curves was calculated and the percentual growth inhibition compared to the positive control of the MRSP test strain was calculated in % (Suppl.Tab.3). All combined treatments resulted in higher growth inhibition than single measures, indicating synergistic interactions. For example, while the single treatment with phage LmqsKl44-4 at an MOI of 0.1 did not inhibit bacterial growth and fusidic acid at 0.25 % resulted in only a slightly reduced growth (19 %), the combined treatment of the two reduced bacterial growth by 75%.
