# Supplementary material for: Bacteriophage and Fusidic Acid Have Synergistic Effect Against Meticillin‐Resistant Staphylococcus pseudintermedius in Ex Vivo Canine Dermis Model
Source: Vet Dermatol. 2025 Sep 18;37(2):200–10. doi: 10.1111/vde.70030 (PMC12967880; doi:10.1111/vde.70030)
Supplement: Supplementary file 3 — Table S1: Characteristics of the study animals. [file VDE-37-200-s003.docx]

SupplTab.1

| **Antimicrobial agent** | **Staphylococcus pseudintermedius** | |
| --- | --- | --- |
|  | **MIC (µg mL^-1^)** | **MIC interpretation** |
|  |  |  |
| Amoxicillin/ Clavulanic acid | 16/8 | R |
| Ampicillin | 32 | R |
| Cefovecin | 8 | R |
| Cephalothin | 32 | R |
| Chloramphenicol | 32 | R |
| Clindamycin | 8 | R |
| Doxycycline | 2 | R |
| Enrofloxacin | 1 | I |
| Erythromycin | 8 | R |
| Florfenicol | 2 |  |
| Gentamicin | 4 | S |
| Oxacillin | 4 | R |
| Penicillin G | 4 | R |
| Pradofloxacin | 0.25 | S |
| Tetracycline | 8 | R |
| Trimethoprim/ Sulfonamide | 4/76 | R |
