# Supplementary material for: Bacteriophage and Fusidic Acid Have Synergistic Effect Against Meticillin‐Resistant Staphylococcus pseudintermedius in Ex Vivo Canine Dermis Model
Source: Vet Dermatol. 2025 Sep 18;37(2):200–10. doi: 10.1111/vde.70030 (PMC12967880; doi:10.1111/vde.70030)
Supplement: Supplementary file 4 — Table S2: Antimicrobial sensitivity test results of the clinical meticillin‐resistant Staphylococcus pseudintermedius . I, intermediate; R, resistant; MIC, minimum inhibitory concentration; S, sensitive. [file VDE-37-200-s005.docx]

Suppl.Tab.2

| Dog | Breed | Age (year/month) | Sex | Reason for euthanasia |
| --- | --- | --- | --- | --- |
| 1 | Bavarian Mountain Hound | 8y | Female intact | Prolapsed intervertebral disc |
| 2 | Labrador Mix | 2y 5m | Male intact | Seizures |
| 3 | Rhodesian Ridgeback | 8y 8m | Female neutered | Tachypnoea/ cachexia |
| 4 | Bordeaux Mastiff | 5y 1m | Male intact | Chylothorax |
| 5 | Labrador Retriever | 8y 7m | Female neutered | Pulmonary hemorrhage/ tachycardia |
| 6 | Husky | 13y 10m | Male intact | Perineal hernia/ rectum prolaps |
