# Supplementary material for: Bacteriophage and Fusidic Acid Have Synergistic Effect Against Meticillin‐Resistant Staphylococcus pseudintermedius in Ex Vivo Canine Dermis Model
Source: Vet Dermatol. 2025 Sep 18;37(2):200–10. doi: 10.1111/vde.70030 (PMC12967880; doi:10.1111/vde.70030)
Supplement: Supplementary file 5 — Table S3: Growth reduction (%) of meticillin‐resistant Staphylococcus pseudintermedius by phage monotherapy or in combination with fusidic acid. MIC, minimum inhibitory concentration; MOI, multiplicity of infection; OD, optical density. [file VDE-37-200-s001.docx]

Suppl. Tab. 3

|  |  | Fusidic acid 0.5 MIC | Fusidic acid 0.25 MIC |
| --- | --- | --- | --- |
|  |  | 85 | 19 |
| Phage LmqsKl44-4 MOI 1 | 27 | 98 | 51 |
| Phage LmqsKl44-4 MOI 0.1 | -1 | 88 | 75 |
